# Towards a Naturalistic Brain-Machine Interface: Hybrid Torque and Position Control Allows Generalization to Novel Dynamics

**DOI:** 10.1371/journal.pone.0052286

**Published:** 2013-01-24

**Authors:** Pratik Y. Chhatbar, Joseph T. Francis

**Affiliations:** 1 Joint Program in Biomedical Engineering at Polytechnic Institute of New York University and State University of New York Downstate Medical Center, Brooklyn, New York, United States of America; 2 Program in Neural and Behavioral Science at State University of New York Downstate Medical Center, Brooklyn, New York, United States of America; 3 Department of Physiology and Pharmacology, State University of New York Downstate Medical Center, Brooklyn, New York, United States of America; 4 The Robert F. Furchgott Center for Neural & Behavioral Science, State University of New York Downstate Medical Center, Brooklyn, New York, United States of America; Weill Cornell Medical College, United States of America

## Abstract

Realization of reaching and grasping movements by a paralytic person or an amputee would greatly facilitate her/his activities of daily living. Towards this goal, control of a computer cursor or robotic arm using neural signals has been demonstrated in rodents, non-human primates and humans. This technology is commonly referred to as a Brain-Machine Interface (BMI) and is achieved by predictions of kinematic parameters, e.g. position or velocity. However, execution of natural movements, such as swinging baseball bats of different weights at the same speed, requires advanced planning for necessary context-specific forces in addition to kinematic control. Here we show, for the first time, the control of a virtual arm with representative inertial parameters using real-time neural control of torques in non-human primates (*M. radiata*). We found that neural control of torques leads to ballistic, possibly more naturalistic movements than position control alone, and that adding the influence of position in a hybrid torque-position control changes the feedforward behavior of these BMI movements. In addition, this level of control was achievable utilizing the neural recordings from either contralateral or ipsilateral M1. We also observed changed behavior of hybrid torque-position control under novel external dynamic environments that was comparable to natural movements. Our results demonstrate that inclusion of torque control to drive a neuroprosthetic device gives the user a more direct handle on the movement execution, especially when dealing with novel or changing dynamic environments. We anticipate our results to be a starting point of more sophisticated algorithms for sensorimotor neuroprostheses, eliminating the need of fully automatic kinematic-to-dynamic transformations as currently used by traditional kinematic-based decoders. Thus, we propose that direct control of torques, or other force related variables, should allow for more natural neuroprosthetic movements by the user.

## Introduction

Planning and execution of motor tasks, such as lifting a cup of coffee, take into consideration the required forces. This becomes apparent when we encounter an object that is much lighter or heavier than expected, making us change our motor strategy to compensate for these newly learned properties of the object. Such properties are called dynamic properties, or properties that take into account the inertia of the object, as well as forces and torques involved in the motion. Kinematics along cannot explain this phenomenon as they take into account the position, velocity and acceleration but not the used or required forces or torques. Additionally, manipulation of the grasped object, such as an egg, requires a relatively narrow range of forces to be applied in order to pick it up without breaking it. The current state-of-the-art BMI prototypes depend exclusively on position or velocity control [1]–[10]. Therefore, currently these BMI algorithms rely on automatic manipulator algorithms to determine the endpoint forces in order to achieve the controlled position or velocity. Thus, fine context-specific control of endpoint forces are beyond the control of most current BMIs [10], a problem that we start to address in the present work.

Movements like waving one's hand in the air vs. water can have the exact same kinematic profiles but require different amounts of forces/torques because of different dynamic properties of the environments. Real-life use of a brain-controlled robotic arm/hand would thus likely benefit from control of dynamic variables like end-point forces and joint torques in addition to kinematic variables. In spite of an established relationship between motor cortical activity and movement dynamics [11]–[18], real-time use of dynamic signals to control a brain-machine interface (BMI) has not been demonstrated. Here we present for the first time a torque controlled BMI in addition to a hybrid joint torque and position controlled BMI using primary motor cortical (M1) spiking activity acquired through high-count microelectrode arrays [19] implanted bilaterally in M1 shoulder and elbow regions [20]. Such a control offers the BMI user direct control over the forces and torques necessary for execution of the movement trajectories. This makes context-specific, precise application of the isometric forces possible.

## Materials and Methods

### Behavioral task

All the behavioral and surgical procedures were approved by IACUC of SUNY Downstate Medical Center and closely supervised and assisted by the Division of Laboratory Animal Resources (DLAR). Two bonnet macaques (*M. radiata*, one female (Subject 1) and one male (Subject 2), weights 3.7–4.0 kg) were trained for this work. The chair training and the task training were based on the principles of positive reinforcement and operant conditioning. No negative reinforcement or aversive stimuli were offered for incomplete/incorrect trials or otherwise.

The subjects were put on controlled water access (CWA) for reaching task training and during the manual and BMI experiments. This regime was in line with the standards established at NIH. Each subject has different water/fluid requirements and cannot be generalized. Such requirements were determined by drinking patterns of the past couple of months. The subjects almost always take the required fluid during the training/experimentation by the water/juice rewards offered. However, it was made sure that the weekly fluid intake is met during this 5 day/week CWA. This was done by offering additional water during off-training days/times in case the subjects did not take the required amount of fluids while performing the behavioral tasks over the training/recording sessions. Any signs of poor nutrition, dehydration or psychological decline were stringently looked for and followed over days and weeks to detect weight-loss. This included daily body weight measurements, urine color, mucosa and skin turgor, abnormal behavior. Provision was made to suspend the training and/or experiment sessions for at least a week should any of these be found and confirmed by a local veterinary doctor as a concern. We did not have to face this scenario with either of our subjects.

**Figure 1:**
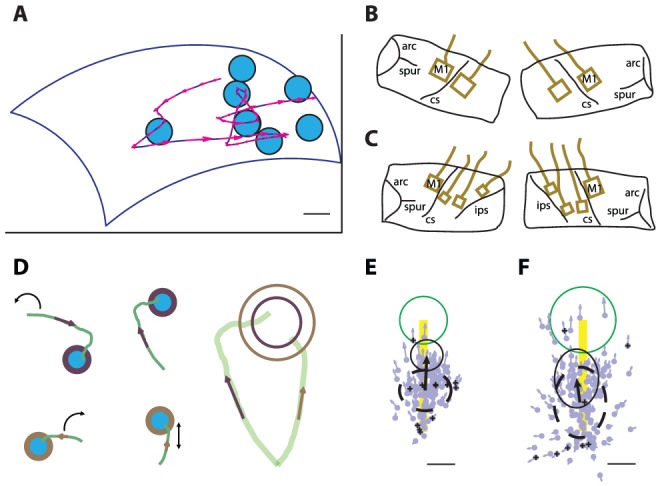
Behavioral paradigm, implant locations and first submovement velocity (FSV) plots. (**A**) Trained bonnet macaques (M. radiata) perform Random Target Pursuit (RTP) task under viscous gain fields, generated by planar exoskeletal robot, with target presentation (light blue filled circles) within pre-defined shoulder and elbow angle boundaries (blue) on the horizontal monitor (edges shown as black lines). Movement trajectory is shown as blue curved continuous line with velocity vectors at different time points of movements as magenta arrows. Scale bar, 2 cm. (**B**, **C**) Chronic microelectrode array implant locations on Subject 1 (**B**) and Subject 2 (**C**) shown by tracing the implantation photographs taken intraoperatively. M1, primary motor cortex; arc, arcuate sulcus; spur, spur of the arcuate sulcus; cs, central sulcus; ips, intraparietal sulcus. (**D**) Schematic of FSV plot generation. Two example movement trajectories are shown and overlaid on top of each other after rotating (curved black arrow) and scaling (vertical arrow in the bottom trajectory schematic) so that the line joining the movement onset and the target center points upwards and is of the same size. The overlay is shown as the panel on the right with both movement trajectories on top of each other. The difference in the size of the targets (shown as open circles of different size) is apparent and is to demonstrate that different scaling was applied on different movement trajectories to match the distance between the movement onset and the target center. For the rest of the panels, only one open circle representing the mean target size of all the normalized trajectories will be shown. FSV vectors for each movement trajectory are shown as colored arrows at the location of peak speed of the first submovement on the movement trajectories (see Methods). (**E**, **F**) FSV plots of example manual task performed by Subject 1 (**E**, n = 118 movements) and Subject 2 (**F**, n = 172 movements). Bold yellow line represents the line joining the movement onset and the center of target. Mean target (after scaling the movement trajectories) is shown as green circle. Thick dashed black circle represents the mean and standard deviation of all FSV locations; thick black arrow and thin black circle represent the mean and standard deviation of FSV vectors, respectively (see Methods). Small light blue arrows in manual task (or small red arrows in BMI task, as shown in **Figure 3**) represent scaled individual FSV and ‘+’ sign at the start of FSV suggest unsuccessful trials. Scale bar, 25% of the bold yellow line.

The subjects were trained on the random target pursuit (RTP) task, where they were required to hit the target that was randomly displayed in the workspace [5]. We used a 2-degree-of-freedom planar robotic manipulandum (KINARM, BKin Technologies, Kingston, ON, Canada) as a right hand exoskeletal system to train these subjects [21]. The target was circular in shape and the radius was 1 or 1.5 cm. The target was displayed in a manner that its center falls inside the workspace spanned by shoulder angles between 10° and 50°–65° and shoulder+elbow angles (i.e., elbow angle in global space) between 85° and 125° (**Figure 1**, **A**). One of the sets of joint angular velocity-dependent (viscous) torque fields was applied and cycled between low and high gains every 10 trials as the subject performed the task (**Table 1**).

**Table 1:** Applied loads on the real or virtual arm as the subject performs the reaching task.

Load set name	Joint velocity-dependent resistive load gains (0.01×Nms/rad)			
	Low load condition (10 trials, small visual feedback cursor)		High load condition (10 trials, big visual feedback cursor)	
	shoulder	elbow	shoulder	elbow
Routine	1	5	4	20
New	2	10	3	15
Equal	5	5	15	15
Increased	10	10	20	20

Once the subject reached the target, s/he was required to stay in the target for 40 ms–160 ms for the trial to be considered successful. On successful completion of a trial, the subject was rewarded with a few drops of water or juice (∼0.25 ml), depending on the subjects preference by a paradigm-controlled juice-reward system (Crist Instrument, Hagerstown, MD). To limit the fluid intake while keeping the subject involved with doing the task for more than an hour, we utilized a random reward schedule: the subject was rewarded randomly on 40% of the correct trials for the manual task. As the subject was newly exposed to BMI task, 100% reward schedule was offered to keep him/her motivated and engaged in the task. The reward schedule for BMI trials was titrated down to 70% depending on the motivation and performance of the subject.

The boundary conditions for the manual task were the physical restrains of the KINARM apparatus, which commonly gives movement range of 30° to 100° at the shoulder angle and 0° to 160° at the elbow angle (local coordinates). For the virtual BMI arm, we set the boundaries to be 5° to 75° for shoulder angle, 0° to 150° for elbow angle and 0° to 160° for shoulder+elbow angle (i.e., elbow angle in global space) with the margins of the visual feedback screen serving as boundary conditions. For an easier task, the boundary conditions were used as the random target generation boundaries plus 5°–10° on each border.

Behavioral data, specifically joint angular positions and torques applied by the KINARM robot on each joint was recorded at 2 kHz sampling rate along with neural recordings (40 kHz for spike detection and unit waveform analysis) on Plexon recording system (Plexon Inc., Dallas, TX). Task specific information was also saved with the same recording system as strobed-word events.

### Surgical implantation

All surgeries were performed under general anesthesia with strict aseptic precautions in a dedicated OR suite. All the standard of care protocols and recommendations were followed. Anesthesia and initial preparations of the surgery were done by an in-house veterinarian personally or under her direct supervision. Pre-operative hydration, NPO and medications (including antibiotics, pain-killers, and inducing agents like ketamine) were administered in conventional fashion. Isofluorane anesthesia was used throughout the surgery. Injectable steroids were used to minimize brain edema and swelling. Mannitol and diuretics like furosemide were kept available if needed. The subjects were given appropriate analgesics, antibiotics and other needed medications by injection throughout the course of surgery and in the post-operative convalescence (commonly 2 weeks). The subjects were observed hourly or two-hourly for the first twelve hours after the surgery by the lab personnel and were examined once or twice daily by the DLAR personnel to quickly detect any potential signs of discomfort.

The subjects were trained on the task with head restraint using a footed headpost (6-FHP-X2F, Crist Instrument, Hagerstown, MD). Implantation was made in the midline slightly superior to the occipital ridge [22]. Head restraint was needed because our experiments also used eye tracking, and neural recordings required several hundreds of electrical connections to the headstage for amplification, of which movement artifacts would saturate the recordings and lead to high loss of information. This also is a common practice in the scientific community and we have not observed any changes in the behavior of the subject or any signs of distress that originate because of the headpost restraint. The subject maintains the same behavior in the cage a few days after headpost implantation and maintains the same level of proficiency and speed of task performance after head restraint.

Once the subject reached the proficiency level of 90% task performance we implanted them bilaterally in primary motor cortex (M1) of shoulder/elbow representative region (**Figure 1**, **B**–**C**). The implantation site was located by intra-operative determination of representative somatosensory cortex using single sharp electrode electrophysiology and then selection of motor cortex adjacent to the area receiving somatosensory inputs from the shoulder region. We used Utah intra-cortical arrays (10×10 electrode grid, 450 µm inter-electrode distance at tip, 1.5 (Subject 1) or 1.0 (Subject 2) mm shank length, Platinum (Subject 1) or Iridium oxide (Subject 2) coating at tip; Blackrock Microsystems, Salt Lake City, UT) [19]. Subject 2 was also implanted bilaterally in primary somatosensory cortex area 1 and 2 of hand representative region and area PE (6×6 electrode grid S1, 1.5 mm shank length, Platinum coating at tip; Blackrock Microsystems, Salt Lake City, UT). Subject 1 was implanted bilaterally in S1 area 1–2 of shoulder and elbow representative region (10×10 electrode grid, 1.5 mm shank length and Platinum-coated tip in left hemisphere, 1.0 mm shank length and Iridium Oxide coated tip in right hemisphere). We used Nesting Platform method to minimize the trans-cutaneous footprint of the implant and reduced the implant-related costs by half [20]. No subject showed any signs of discomfort at or around implantation site. The connectors mounted on the Nesting Platform offered an easy, pain or discomfort free way of connecting the microelectrode arrays to the recording hardware.

Subject 1 was implanted for the third time in contralateral (left) M1 and S1 cortices with 1.5 mm shank length arrays (previous two implantations also covered dorsal premotor cortex), but we were not successful with the neural recordings from these regions with the current implant. This might be because of previous surgical insults to the gray matter and/or consequent healing and gliosis, preventing the close contact of the array tips with the neurons. Right sided S1 array for Subject 1 (fresh implant) also failed to record any spiking neural activity, possibly due to high wire-bundle strain and eventual spontaneous explantation of the array post-operatively. We used neural recordings from the right-hemisphere (ipsilateral) M1 cortex from Subject 1 and left-hemisphere (contralateral) M1 cortex from Subject 2 for closed-loop experiments. Thus, this work also provides evidence for an Ipsilateral closed-loop BMI.

### Neural recordings

After implantation surgery, the subjects were allowed to recover for 2–3 weeks after which recordings of single unit activity were taken while the subject performed the reaching task. Recordings were made using externally synched multiple multichannel acquisition processor systems [6] (MAPs, Plexon Inc., Dallas, TX). Neural signals were amplified, band-pass filtered (170 Hz–8 kHz), sampled at 40 kHz, thresholded and single/multiple units were sorted based on their waveforms using principal-component-based methods in Sort-Client software (Plexon Inc., Dallas, TX). Neural spike timestamps were streamed online using TCP/IP protocol through PlexNetConc (Plexon Inc., Dallas, TX) to the computer where spike-based-predictions are made based on previously calculated weights from manual task. EMG recordings (bilateral pectoralis major, latissimus dorsi, anterior and posterior heads of deltoid, biceps brachii, triceps brachii) using surface electrodes (Grass Technologies, West Warwick, RI) at 2 kHz sampling rate were made during the task performance. Under BMI task, we did not find significant correlations between EMG activity and cursor movements under both locked and moving arm condition, suggesting that proprioceptive signals or motor signals controlling muscle activity were not contributing to the BMI performance. Eye tracking was performed under some experimental sessions to confirm that the subject is paying attention to the workspace and the task in general, but was not used for any further analysis.

### BMI algorithm

The torques generated by the subjects were calculated using equations of inverse dynamics [16], [17], [21]. We estimated the inertial properties of the limb segments using linear regression equations based on limb segment lengths and subject weight [23] and those of the robotic systems were made available by the company that commercializes KINARM (BKin Technologies, Kingston, ON, Canada). The generalized form of such inverse dynamics equation can be described as:
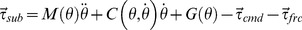
(1)where 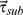 is the torque generated by the subject (with separate shoulder and elbow components), 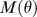 is the inertial matrix of the whole system, 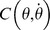 is the term for coriolis and centripetal forces, 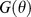 is the gravity term (which will be 0 in our case because of the planar nature of the manipulandum performing movements about the horizontal plane). 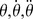 denote joint (in our case, shoulder and elbow) angular positions, velocities and accelerations, respectively. 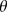 and 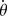 are sampled at 2 kHz and then low-pass filtered at 10 Hz using 6-pole (3 pole forward, 3 pole backward) Butterworth filter. 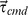 represent commanded torques by the attached motors in order to create the virtual environment. 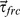 represent friction torques generated inside the torque motors as the subject makes the movements, which were calculated as tanh function of the product of the joint angular velocities and static friction parameters, as supplied by BKin technologies. The negative sign before the last two terms signify that the subject has to overcome those torque values in order to make the movements.

**Figure 2:**
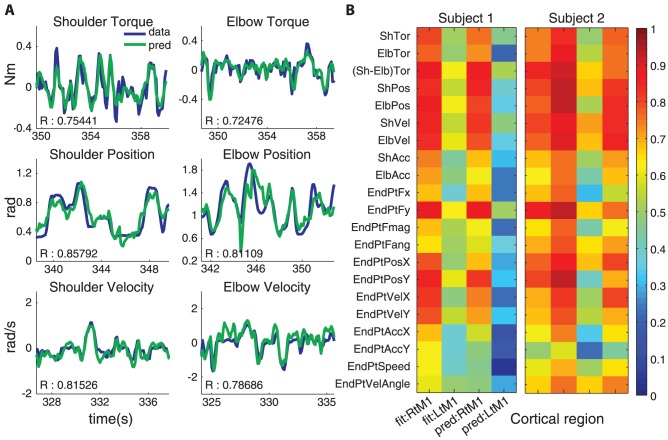
Open loop performance of the decoder. (**A**) Sample plot of prediction values (green) overlaid on recorded positions, velocities or calculated torque values (blue) during manual task. R, correlation coefficient. (**B**) Summary R value plots of fits (estimates on the same dataset from which the multiple linear regression coefficients were calculated, left 2 columns) and predictions (estimates on the new dataset, right 2 columns) for ipsilateral and contralateral motor cortical signals on multiple behavioral variables. Note the comparable accuracies among dynamic (e.g., torque) and kinematic (e.g., position, velocity) variables. Acronym key: fit, fit values; pred, prediction values; Rt, Right; Lt, Left; M1, primary motor cortex (shoulder and elbow representative region); Sh, shoulder; Elb, elbow; Tor, torque; Pos, position; Vel, velocity; Acc, acceleration; EndPt, End-point/end-effector – located about the tip of middle finger of the subject; F, force; X, X-direction; Y, Y-direction; mag, magnitude.

We used first 20 principal components of the spiking neural activity of past one second binned at 100 ms interval (10 bins) to predict movement kinematics and dynamics [17]. The performance of this decoder in offline or open loop mode is shown in **Figure 2**. The general form of such a decoder is described below:
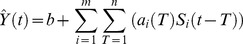
(2)where 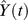 is the predicted variable of interest (e.g., torque, position) at time 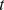; 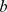 is the y-intercept, 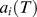 are the filter coefficients for the 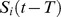 that represent the score of the 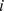
th principal component of the population spike rate at time bins 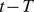 (we used 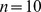 and 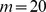 as described above). These filter coefficients were derived with multiple linear regression methods from the data collected from the manual task. The transformation of neural activity space to principal component space can be described in matrix notation by:
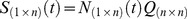
(3)where 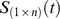 is the vector of principal component scores for a given time bin 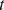; 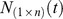 is the vector of normalized neural spike counts on each unit for the same time bin 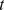, and 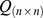 is the principal component coefficient matrix that was calculated from about 3–5 minutes of the spiking neural activity at the beginning of the recording session or from one of the previous sessions.

Predicted shoulder and elbow joint torques and angular positions (total four variables) were sent to the behavioral paradigm plant. Behavioral paradigm plant is a mathematical representation of the subject's right arm with exoskeletal robotic manipulandum. In our case, the plant was running at a rate of 1 kHz (plant update interval 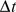 of 0.001 seconds) on xPC target (Mathworks, Natick, MA). The joint angle position values of the plant running at discrete sufficiently small sampling intervals 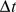 will be,
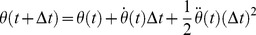
(4)Where 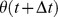 are the joint angular position values at time 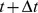 (next time-step of the plant), which depend on the current joint angular positions 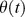 and joint angular velocities 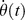 of the plant as well as the joint angular accelerations 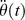 resulting from the applied forces/torques to the plant at time 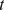. In case of our hybrid torque-position BMI, the acceleration is given as a weighted sum of the joint angular accelerations as a result of joint angular position predictions 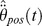 and the joint torque predictions 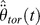.
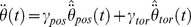
(5)

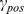
 and 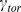 in Equation 5 are the coefficients that determine, or scale, the influence of joint angular accelerations resulting from the joint angular position and torque predictions, respectively, on the hybrid BMI. Inserting the value of Equation 5 in Equation 4 will give,
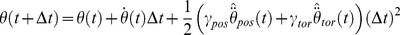
(6)
Equation 6 is the hybrid torque-position control that we used in this work. The joint angular accelerations caused by the joint torque predictions, 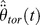, are straight-forward to calculate using the following formula,
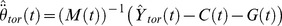
(7)Where the neural predictions for the joint torques are defined as 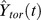. 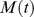, 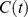 and 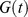 are the inertial, coriolis and gravitational terms of the plant at time 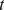. Because of the horizontal planar configuration of our setup, 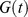 will be 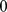 in our case as previously mentioned. Update of the plant joint angular positions in a special case of pure torque control mode (no influence of joint angular position predictions) can be done by,
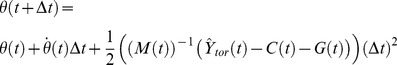
(8)Thus pure torque control shown in Equation 8 with our hybrid torque-position control equation (Equation 6) is achieved by using 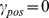, 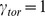 and inserting the values from Equation 7 in Equation 6. However, special case of pure position control would be achieved if the plant position at time 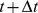 matches the neural predictions of the joint angular positions 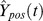 provided to the plant, i.e.,
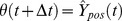
(9)Neural predictions of the joint angular positions, 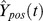, are used as final estimates of 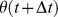 and not the difference between the actual and predicted joint angular positions. Therefore, it is not possible to come up with 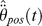 by taking double time-differential of 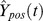. We derived 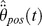 that satisfies the pure position control shown in Equation 9 with our hybrid torque-position control shown in Equation 6. Inserting values from Equation 9 in Equation 6 as well as using 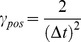 and 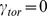 yielded,
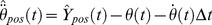
(10)The second term on the right 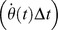 is a single time-step integral of joint angular velocities. This is to update the changes in the joint angular positions as a result of the joint angular velocities in the plant. Note that 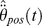 as defined in Equation 10 is a practical workaround and not the actual acceleration derived from position predictions. For the hybrid torque-position BMI results presented here, 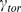 was set to 1 or very close to 1 and the values of 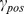 were varied between 0 and 200 in Equation 6.

### First Submovement Velocity (FSV)

In general a reaching movement can be decomposed into multiple submovements. The very first sub movement can be considered the output of a feedforward controller, while subsequent submovements can be considered to be the output of a feedback mechanism. In order to compare the feedforward component of the reaching movements made under manual control and those made under BMI control we studied the first submovement. To this end we compared velocity information at the peak speed of the first submovement, including not only the magnitude of this peak speed but also the direction of motion at it. Briefly, submovements in a given movement profile are characterized by the occurrence of two jerk zero-crossings that flank the peak speed [24]. Jerk is the third time-derivative of position, after speed and acceleration, which are the first and second time-derivatives of position, respectively. In acceleration space, the peak speed would be a zero-crossing.

### Finding the movement peaks

Shoulder and Elbow angular position data was collected at 2 kHz sampling rate. It was low-pass filtered at 10 Hz using a 6-pole (3 forward, 3 backward) Butterworth filter. End-point position was then calculated using trigonometry and numerical differentiation to come up with end-point velocities and speed. Peaks and troughs of the speed were then found using calculus methods previously described [24]. Trials meeting the following criteria were considered for the analysis: (i) peak values of the speed higher than 5 cm/sec, (ii) start of the movement (trough preceding the peak) at least 100 ms after the presentation of the target (iii) peak of the speed at least 200 ms after the presentation of the target and finally (iv) start of the movement point is at least 1 cm away from the margin of the target in workspace.

### Normalizing the movements

All included trials are normalized by rotating and scaling the movements with respect to start-to-target vector. Start-to-target vector is defined as the line joining the point of the movement onset and the center of the target. First, the movement trajectory was isolated from the movement onset to the end of the trial. This movement trajectory was then rotated such that the start-to-target vector for a given movement trajectory points upwards. The movement trajectory was then scaled such that the length of start-to-target vector is uniform over all the movement trajectories. Notice that this length in reality is always greater than 1 cm plus target radius (making total of at least 2–3 cm) considering the inclusion criteria that we have used, and can go as high as 15 cm in cases of targets presented at the extremes of the workspace. This normalized movement trajectory was then broken down into submovements by analyzing the temporal differentials of the speed, namely acceleration and jerk [24]. The first or primary submovement was used for analysis and the velocity at the peak speed was deemed to be representative of the first submovement. We located the FSV on the workspace and calculated perpendicular and parallel components as a fraction of the distance between the point of movement onset and the target center (**Figure 1**, **D**).

### FSV locations, vectors, plot legends and statistics

**Figure 3:**
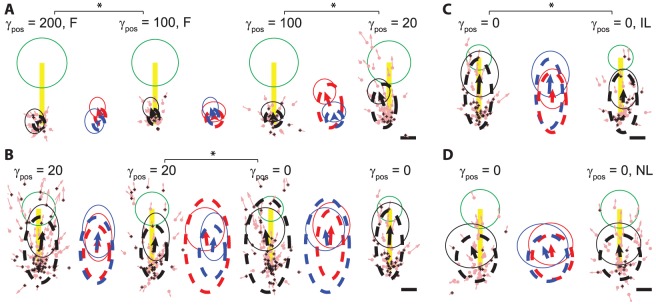
Effect of position influence and environmental changes in hybrid torque-position BMI. (**A**) Decreasing position influence (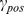, see methods) from 200 (n = 32) to 100 (n = 46; 42) to 20 (n = 75) and (**B**) from 20 (n = 127; 92) to 0 (pure torque control; n = 116; 77) moved the FSV locations away from the movement start-point and towards the target (*P*<0.05), indicating more ballistic movement profiles. (**C**) Increased viscous gain-fields (Increased Load, IL, see **Table 1**; right; n = 74) from before (Equal Load, see **Table 1**; left; n = 77) moved the FSV locations towards the movement start-point and decreased the FSV vector lengths (*P*<0.05), indicating decreased movement velocities under high resistance. (**D**) Introduction of new viscous gain-fields (New Load, NL, see **Table 1**; right; n = 81) within the bounds of ongoing low- and high- gain fields (Routine Load, see **Table 1**; left; n = 76) led to no statistically significant differences in the FSV locations or vectors. Same conventions as in **Figure 1**, **E**, **F** are used. Means and standard deviations of the FSV locations and vectors of different sessions (mean FSV location: base of arrow; standard deviation of FSV location: dashed circle, scaled 10%; mean FSV vector: arrow itself; standard deviation of FSV vector: thin solid line circle, scaled 10%) are overlaid in between the FSV plots for easy comparison; blue: from the FSV plot on the left, red: from the FSV plot on the right. The arm was restrained at the bottom left (75° shoulder and 85° elbow angles, **A**, **D**) or at the center (25° shoulder and 85° elbow angles, **B**, **C**) of the workspace unless indicated as F, free or unrestrained arm.

The FSV vector is defined as the velocity vector at the peak speed of the first submovement on the normalized workspace. The FSV location is defined as the location of this peak speed on the normalized workspace. Normalized start-to-target vector is plotted as vertical yellow bar of unitary length and mean normalized target size is plotted as a green circle. Individual FSV locations are plotted as blue or red dots in case of manual or BMI task, respectively. Similarly, individual FSV vectors are plotted as blue or red arrows in case of real arm or virtual arm movements, respectively (scaled to 1% of original). Unsuccessful trials are marked by black plus (+) signs on individual FSV locations. The mean and standard deviation of FSV locations is plotted as dashed circle/oval with the mean FSV location at its center and standard deviation as its margin. The mean and standard deviation of the FSV vectors is plotted as solid arrow and thin circle respectively (scaled to 10% of original). The mean and standard deviations of FSV locations and vectors of sessions to be compared were overlaid for ease of visualization in **Figure 3**. We used non-parametric Kuiper two-sample test statistic (circular statistic analogue of Kolmogorov-Smirnov two-sample test) to compare the distributions of angles of FSV locations and vectors. To compare the distribution or lengths of FSV locations and vectors, we used non-parametric Kolmogorov-Smirnov two-sample test statistic.

## Results and Discussion

Two bonnet macaque (*M. radiata*) subjects performed a planar random target pursuit (RTP) task (**Figure 1**, **A**) using a right-handed exoskeletal robotic system [21]. Towards separating the neural signatures of kinematic and dynamic variables, they were exposed to high and low resistive viscous force field environments (cycled in blocks of 10 trials, See **Table 1**). This can be compared with moving in air (low viscosity) or water (high viscosity). The subject-generated torques were calculated using equation of inverse dynamics as described in Equation 1 [16], [17], [21], which uses inertial estimates of the exoskeletal robot and the limb segments [23]. We did not notice significant task-related movements or EMG activity in their left upper limbs in either manual or BMI tasks. We reconstructed a variety of kinematic and dynamic-related parameters with a multiple linear regression method as described in Equations 2 and 3 (**Figure 2**, **A**) [17]. We used correlation coefficient (R) between the measured and predicted behavior as a means to quantify prediction accuracies across a variety of behavioral parameters and confirmed that both kinematic and dynamic variables have comparable fit and prediction accuracies (**Figure 2**, **B**). Superior fit/prediction performance of ipsilateral M1 neural activity in Subject 1 was most likely due to poor electrode recordings in the contralateral M1, which had been previously implanted several times. This further bolsters the previously documented findings [9] that the ipsilateral cortex can also be used to derive both kinematic and dynamic information for the BMI performance in case of contralateral cortical damage or unavailability.

Under BMI control mode, reconstructions of a single behavioral parameter (joint torques or joint angular positions) or any scaled combination of the two were fed continuously to the BMI plant (Equation 6) to come up with the final cursor position. Inclusion of torque predictions in closed-loop control leads to more naturalistic movement profiles (**Video S1, S2, S3**) when compared with pure position control (Equation 9) without or with smoothing (**Video S4** and **Video S5**, respectively). Open loop control while the subject was performing manual task (similar to offline predictions) demonstrated that the hybrid torque-position control was efficiently predicting the movement trajectory (**Video S1**). Under brain-control mode, the subject was able to successfully perform the task with such hybrid torque-position control in a closed-loop (**Video S2**). To quantify the feedforward aspects of the movements under brain-control mode, we used the first submovement velocity (FSV, **Figure 1**, **D**) as our error proxy. When the subject uses her/his arm to make natural ballistic movements, the mean FSV location (center of dashed circle) is approximately at a point halfway on the line joining the movement start point and the target and mean FSV vector (arrow) points towards the target (**Figure 1**, **E**, **F**). Under hybrid BMI control, decreasing the influence of position control in the hybrid controller moved the FSV locations towards the target and further away from the movement start-point (**Figure 3**, **A**–**B**, *P*<0.05). On switching to pure torque control (i.e., no position predictions contribution to drive the BMI, Equation 8), the movements became more ballistic (**Video S3**). The angle of mean FSV location and direction changed (*P*<0.05, Kuiper test) and distribution of FSV location expanded (**Figure 3**, **B**, *P*<0.001, K-S test). The change in the mean FSV location and vector away from the movement start-point under higher relative influence of torque control can be seen as increased admittance/compliance or decreased impedance. Thus by varying the amounts of relative influences of torque and position in the hybrid BMI controller one can get a handle on the stiffness or impedance of the prosthetic arm.

We employed pure torque control (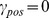, Equation 8) to test the influence of external dynamic environments on the BMI behavior in closed-loop. Absence of position/kinematic control ensured that the dynamic properties of the environment affect the BMI behavior to the fullest. Introducing increased viscosity environments (from equal to increased, see **Table 1**) moved the FSV locations near the movement start-point with shorter FSV vector lengths (**Figure 3**, **C**, *P*<0.05, K-S test). This indicates that the BMI movements slowed down when the external environment exerted higher resistance. On the other hand, introducing new viscous gain fields within the bounds of low and high viscous fields that the subject is currently experiencing (from ‘Routine’ to ‘New’, see **Table 1**), we did not find significant differences in the FSV locations or vectors. (**Figure 3**, **D**). Note that the changed behavior of torque control BMI under increased viscosities can be explained by facilitated direct interaction of the user with the dynamic properties of the environment. Such phenomenon cannot be observed under pure kinematic control because the automatic kinematic-to-dynamic algorithm would overcome the increased viscous loads involuntarily towards maintaining user-instructed kinematic goals.

In the natural acts of reaching and grasping, we routinely increase the stiffness of the proximal joints (shoulder, elbow) towards providing stability to the distal links (wrist and hand/finger joints). In order to fully exploit this functionality of hybrid BMI control, the relative influence of position (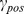) and torque (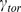) predictions on the individual joints needs to be controlled using the neural signals, which we leave for a future study. Many refinements for superior BMI performance have been previously documented. Towards building smart adaptive decoders, principles of reinforcement learning [2], [25]–[27], coadaptation [28] and feedback control design [29] have been implemented. Inclusion of cognitive/behavioral states (awake/alert, asleep, task-focused, distracted etc.) has been proposed towards robust decoding of neural signals for prosthetic arm control [30]. Incorporation of sensory feedback is shown to enhance BMI control [31] and intracortical microstimulation has been used to provide tactile feedback in brain-machine-brain interfaces [32]. Our results suggest that real-time control of joint torques gives the user a more direct handle on movements under changed dynamic environments. Inclusion of torque predictions in BMI algorithms can play a crucial role in sensorimotor neuroprostheses, as the direct neural control of torques at each joint of the prosthetic device will bring us closer to the ultimate goal of majority of the dexterous movements reaching and grasping.

## Supporting Information

Video S1
**Manual task overlaid with offline predictions show robust decoding of movement trajectories.** Subject is performing random target pursuit (RTP) task using the right upper limb with mounted planar exoskeletal robot. Black tracings with white circular visual feedback cursor at the end-point represent the actual arm. Origin point at around (5,−15) represents the shoulder location and first angle represents the elbow location. Light blue arm with a diamond-shaped visual feedback cursor at the end-point is the simulation of offline predictions of the virtual arm using the past one second of neural activity of M1. The small or large size of the visual feedback cursor at the end-point indicates the low or high load environment, respectively. 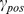 was set to 20. The subject was not given any visual or somatosensory feedback of these reconstructions while doing the manual task. Yellow color of the visual feedback cursor indicates that the subject is looking away from the screen (screen margin represented by a dark blue line, bottom right corner of which is visible; yellow color for the purposes of this video only – the actual task shows white circle throughout the task for visual feedback purposes). Task execution time (in seconds) is displayed on the top left corner. Big or small cursor size represents high or low viscous loads (see Table 1) on the arm, as applied by the KINARM motors. Note that the virtual arm reconstructions match closely with the real arm movements and do carry a ‘projectile’ component of the movement as occurs in natural movements. Our algorithm does not use or need heavy filtering or smoothing of the predictions, partly because the torque predictions automatically get ‘filtered’ by the equations of dynamics containing inertial properties of the virtual limb.(AVI)

Video S2
**Hybrid BMI task with torque and position control offers natural movement profiles.** Neural control of the virtual arm with 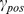 set to 20. The actual arm was locked to 25° at shoulder angle and 85° at elbow angle to discourage contribution of neural signatures of any proprioceptive inputs towards the task execution. The subject “closes” the BMI loop by means of visual feedback. Here, the virtual arm is visible by the subject as a diamond-shaped end-point visual feedback cursor on the computer screen. The visual feedback of the real arm is turned off (i.e., white circle is invisible to the subject). Big or small cursor size represents high or low load on the virtual BMI arm. For the purposes of this video only, the cursor color is changed to yellow when the subject looks away from the screen. Actual task keeps the same light blue cursor color throughout the task. Other conventions are same as noted in Video S1. Note the resemblance in the movement trajectories between the closed-loop BMI control presented in this video and the open-loop predictions shown in Video S1.(AVI)

Video S3
**BMI task using pure torque control leads to more ballistic movements.** Neural control of the virtual arm with 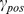 set to 0. Note more expansive, less restrained movements as echoed in the larger distribution of FSV locations and vectors on Figure 3, B. Video generated using the same conventions as used in Video S2.(AVI)

Video S4
**BMI task using position control offers less natural and jittery movements.** Neural control of the virtual arm with limb joint position predictions only (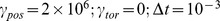). No smoothing or filtering of the position predictions was performed. Note jittery, less ballistic and sub-naturalistic movements. Video generated using the same conventions as used in Video S2, except that the visual feedback cursor color is white.(AVI)

Video S5 **BMI task using filtered position predictions lead to smooth but slower movements.** Neural control of the virtual arm with limb joint position values that are estimated by averaging past 10 pure joint position prediction values over 50 ms window. Note that now the movement profiles are smooth but slower and less spontaneous when compared with pure position control without smoothing. Video generated using the same conventions as used in Video S4.
(AVI)
